# Comparing HIV risk-related behaviors between 2 RDS national samples of MSM in Brazil, 2009 and 2016

**DOI:** 10.1097/MD.0000000000009079

**Published:** 2018-05-25

**Authors:** Mark Drew Crosland Guimarães, Carl Kendall, Laio Magno, Gustavo Machado Rocha, Daniela Riva Knauth, Andrea Fachel Leal, Ines Dourado, Maria Amélia Veras, Ana Maria de Brito, Ligia Regina Franco Sansigolo Kerr

**Affiliations:** a Department of Preventive and Social Medicine, Federal University of Minas Gerais, Belo Horizonte, Brazil; b Center for Global Health Equity, Tulane School of Public Health and Tropical Medicine, New Orleans, LA, USA; c Department of Life Sciences, State University of Bahia, Salvador, Bahia; d Federal University of São João Del-Rei, Divinópolis; e Federal University of Rio Grande do Sul, Porto Alegre; f Collective Health Institute, Federal University of Bahia, Salvador, Bahia; g Faculdade de Ciências Médicas da Santa Casa de São Paulo, São Paulo; h Aggeu Magalhães Institute–FIOCRUZ, Recife; i Federal University of Ceará, Department of Community Health, Fortaleza, Ceará, Brazil.

**Keywords:** behavior surveillance, Brazil, HIV/AIDS, MSM

## Abstract

**Introduction:**

Periodic monitoring of sociobehavior characteristics at a national level is an essential component of understanding the dynamics the human immunodeficiency virus (HIV) epidemic worldwide, including Brazil.

**Methods:**

This paper compares descriptive sociobehavior characteristics in 2 national cross-sectional HIV biological behavioral surveillance surveys (BBSS) conducted in 2009 and 2016 among men who have sex with men (MSM) in Brazil. Respondent driven sampling (RDS) was used for recruitment in both years. Overall proportions were weighted according to Gile's estimator using RDS Analyst Software and 95% confidence intervals were calculated for comparisons between the 2 periods. Further comparisons were stratified by age groups (<25 and 25+ years old).

**Results:**

Overall, 3749 and 4176 MSM were recruited in 2009 and 2016, respectively. In 2016, participants were younger than 25 years old (58.3%), with 12 or more years of education (70.4%), with higher socioeconomic status (40.7%), and had a higher proportion of whites (31.8%), as compared to 2009. Also, participants in 2016 reported less alcohol use and binge drinking, but used illicit drugs more frequently. There was an increase among MSM who self-reported their HIV risk as low and had low HIV knowledge while the proportion of those who were never tested for HIV dropped from 49.8% in 2009 to 33.8% in 2016. Although more than three-quarters received free condoms in both years, STD counseling remained low (32% and 38% for 2009 and 2016, respectively). Sexual risk behavior remained at high levels, especially unprotected anal receptive sex and sex with multiple partners. Younger MSM (<25 years old) showed riskier sexual practices than those 25+ years old, when comparing 2016 to 2009.

**Conclusions:**

Our results indicate a worrisome risk behavior trend among Brazilian MSM, especially among younger ones. These results can contribute for a better understanding of the HIV epidemics in Brazil, with timely shift in strategies so improved effectiveness in public health prevention efforts can be achieved.

## 1 Introduction

Despite trends in decline of new human immunodeficiency virus (HIV) infections and deaths worldwide,^[1]^ recent data from Brazil indicate a high prevalence of HIV among men who have sex with men (MSM)^[2,3]^ and a worrisome increase in mortality rates in some regions, such as north and northeast.^[4]^ The HIV/acquired immune deficiency syndrome (AIDS) epidemic continues to grow disproportionately among MSM worldwide.^[5]^ It is estimated that MSM are almost 20 times more likely to be HIV infected compared to the general population in low- and middle-income countries.^[6]^ Even in high-income countries HIV epidemic is reemerging among MSM as a serious public health problem.^[7]^

Biological factors, including higher susceptibility of infection through unprotected anal sex,^[8]^ and behavioral characteristics, for example, high proportion of unprotected sex and multiple sexual partners,^[9]^ in association with social and structural factors, such as stigma, discrimination and lack of or poor access to prevention programs,^[7]^ may help explain this disproportionally burden of HIV infection among MSM. This trend is of special concern among young (<25 years old) MSM worldwide,^[10–13]^ including Brazil.^[14]^

Although Brazil was one of the first countries to successfully introduce free access to treatment and prevention actions for all, long-term sustained effectiveness may be jeopardized by controversial public health policies, including a recent national restriction on health education programs which target young MSM, a reduction in the number of and funding for HIV/AIDS nongovernmental organizations (NGO), and zero funding to reduce stigma and discrimination.^[15]^ Unintentionally, the strong emphasis on medicalizing the response to the epidemic may undermine prevention.

Interpretations of trends in HIV infection among key population groups should be preceded by an exploration of contextual factors, including social, cultural, and behavioral changes. These changes, along with changes in the prevalence of HIV and other sexually transmitted infections (STI), are monitored by periodic biological and behavioral surveillance surveys (BBSS) as recommended by various national and international agencies, including World Health Organization (WHO), United Nations Program on HIV/AIDS (UNAIDS), and Centers for Disease Control (CDC).^[16,17]^ Monitoring both, HIV prevalence and risk-related behaviors, in repeated surveys was key to the development of a world-wide UNAIDS evaluation system as programmatic efforts grew at the end of the 20th century.^[18,19]^ Brazil has been conducting surveillance surveys for HIV in key populations in cities throughout Brazil since the early 1990s, and formalized this practice in a nation-wide 10-city BBSS using RDS in 2009.^[2,20,21]^ While the logic of BBSS demands comparison across time and interpretation of trends, this has rarely been accomplished.

Periodic monitoring of sociobehavior characteristics at a national level may contribute to assess the impact of HIV/AIDS strategies adopted by countries worldwide. Brazil, through its Department of STI, HIV/AIDS and Viral Hepatitis, Ministry of Health (DIAHV/MoH), has accordingly decided to carry out periodic BBSS focused on selected population groups, including MSM, female sex workers, and, more recently, transgender population.^[2]^ The aim of this paper is to compare risk-related behaviors across the 2 nation-wide samples using RDS conducted among MSM.

## 2 Methods

### 2.1 Study population

This is a descriptive analysis derived from 2 national cross-sectional BBSS among MSM in Brazil, in 2009 and 2016. Men who reported anal or oral sex with other men in the previous 12 months were recruited using respondent driven sampling (RDS) methodology.

Detailed procedures have been previously described.^[3,22]^ Briefly, following formative researches, from 5 to 8 MSM were chosen to start the recruitment process. Each one received 3 numbered coupons for distributing to their peers. Eligibility criteria included those who were 18 years old or over, who self-reported sex with another man in the previous 12 months, who lived, worked or studied in each host city, and received a valid coupon from a peer. Written informed consent was obtained from each eligible participant.

The DIAHV/MoH identified 10 cities in 2009 and 12 cities in 2016 for the study. Eight cities participated in both BBSS (Belo Horizonte and Rio de Janeiro–Southeast region; Brasília and Campo Grande–Central-west region; Manaus–North region; Recife and Salvador–Northeast region; and, Curitiba–South region). Santos (Southeast region) and Itajaí (South region) were included in 2009 only, while Belém (North region), Fortaleza (Northeast region), Porto Alegre (South region), and São Paulo (Southeast region) were added in 2016. The 2 cities were excluded due to low participation rates and several other operational factors. The 4 cities were added to increase representativeness of sites in their regions.

### 2.2 Data collection

Computer-assisted personal interviews were conducted in both 2009 and 2016 surveys. Participants were interviewed by trained staff, or if desired, were able to respond directly on the tablet for the 2016 survey, after proper orientation. The 2016 questionnaire was based on the 2009 survey—itself based on previous UNAIDS BBSS—in order to provide sound comparisons. Detailed information on the questionnaires and other procedures are provided in previous publications.^[3,22]^ Briefly, both questionnaires were similar and included data on: eligibility and social network characteristics; socioeconomic and demographic information; HIV/AIDS knowledge, access to health services, including HIV prevention and treatment; history of testing for HIV, syphilis and hepatitis; sexual identity, visibility, violence, stigma and discrimination; and risk behavior, including unprotected sex, illicit drug, and alcohol use. For the 2016 survey, information on HIV post- and preexposure prophylaxis as well as HIV/AIDS treatment were added. In addition, those who wished were tested for HIV and syphilis in both years. In 2016, hepatitis B and C tests were added and standard rapid tests (RT) as recommended by the DIAHV/MoH were used. RT were performed on peripheral blood drawn from each participant in 2016 and on samples obtained from finger stick in 2009. For both years, pre- and posttest counseling were carried out and those with any positive results were referred for medical follow-up.

For this analysis, we focused on selected sociodemographic and behavior characteristics, with emphasis on sexual behavior. Age was categorized as <25 or 25+ years old, schooling as <12 or 12+ years of formal education, race as white or nonwhite. Socioeconomic class was classified according to Brazilian Criteria as A/B (higher) or C/D/E (lower).^[23]^ Substance use variables were alcohol use (>4 or ≤4 times a month), binge drinking (4 or more drinks in one sitting taken less than or more than once a month), and any illicit drug use, within the last 6 months. Sexual identity, discrimination, physical, and sexual violence due to sexual orientation were also included in this analysis.

Sexual behavior indicators analyzed were age of sexual debut (<15 or 15+ years old), condom use in the first sex, number of partners, unprotected anal receptive or insertive sex, exchange of sex for money, stable partnership, sex with men only, and use of virtual media or physical locations for finding sex partners (cruising). The last 8 indicators were assessed in the previous 6 months.

Finally, indicators of HIV knowledge, self-reported HIV risk, sharing HIV prevention information with friends, previous HIV testing, and receiving free condoms and STI counseling in the last 12 months were also assessed. HIV knowledge was based on questions recommended by DIAHV/MoH and UNAIDS and were analyzed using item response theory (IRT) as previously described.^[24]^ Knowledge scores were categorized according to percentile distribution as follows: <25%, 25–75%, and >75%, as low, moderate, or high knowledge score, respectively.^[25]^

### 2.3 Statistical analysis

The main objective of this analysis was to descriptively compare the 2 BBSS surveys, using aggregated data for each year. The proportion of the selected characteristics was estimated for each year, separately, using complex sample analysis in order to take into account the sampling design, in which each city was treated as a stratum. Gile's successive sampling estimator^[26]^ was used to generate weighted estimates using RDS Analyst Software (Version 0.57) for each city.^[27]^ Change in estimates was assessed by their respective 95% confidence intervals (CIs), indicating whether they overlapped between the 2 periods or not.

We initially compared the overall proportions between 2009 and 2016. For selected indicators we stratified both BBSS surveys by age groups (25+ and <25 years old), and the weighted proportion were compared with regard to their magnitude and direction from 2009 to 2016. Finally, for 2016 we compared the weighted proportions of selected indicators among the 12 cities in order to describe potential heterogeneity. All the analyses were carried out using SAS Statistical Package complex survey procedure (Proc SurveyFreq, Version 9.4). Both studies were approved by the Committee on Research Ethics of the Federal University of Ceará (UFC), credentialed by the National Commission on Research (CONEP #14494 and #1.024.053, for 2009 and 2016, respectively).

## 3 Results

The current analysis included 3749 and 4176 recruited MSM for, respectively, 2009 and 2016 studies. Compared to 2009, the 2016 study population was younger than 25 years old (38.3% vs 58.3% for respectively, 2009 and 2016), with 12 or more years of schooling (27.8 vs 70.4%), higher socioeconomic class (24.4% vs 40.7%), and with a higher proportion of white race (16.3% vs 31.8%) (Table 1 ). Similarly, in 2016 more participants identified themselves as gay/homosexuals (59.3% vs 83.1%), but also reported more discrimination (27.1% vs 64.6%), physical (12.8% vs 23.9%), and sexual violence (14.9% vs 20.9%). While an increase in the proportion of illicit drug use in the 2016 survey was observed (42.6% vs 48.4%, for respectively, 2009 and 2016, CI overlapping), there was a decrease in alcohol use more than 4 times a month (34.6% vs 24.2%) and binge drinking more than once a month (47.4% vs 27.3%). As shown, CI for the latter 2 indicators did not overlap.

**Table 1:**
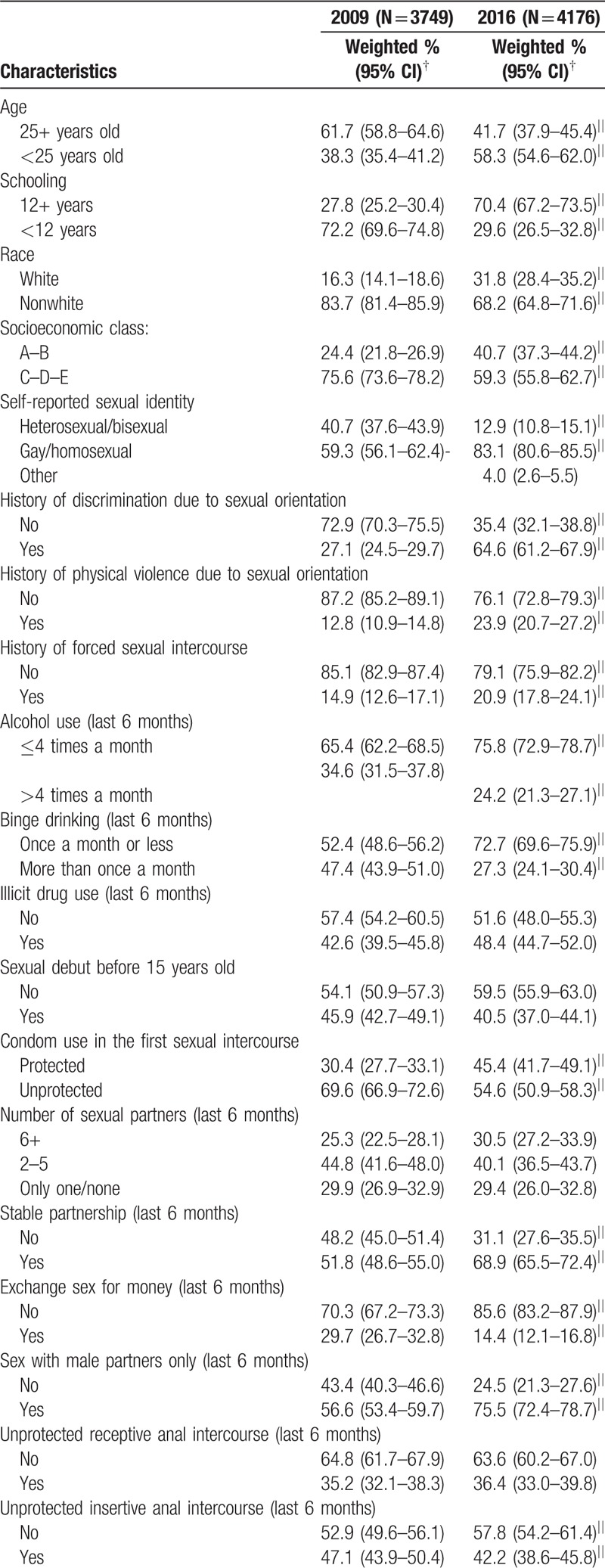
Selected sociobehavior characteristics among 2 cross-sectional MSM RDS studies in Brazil^∗^, 2009 and 2016.

**Table 1 (Continued):**
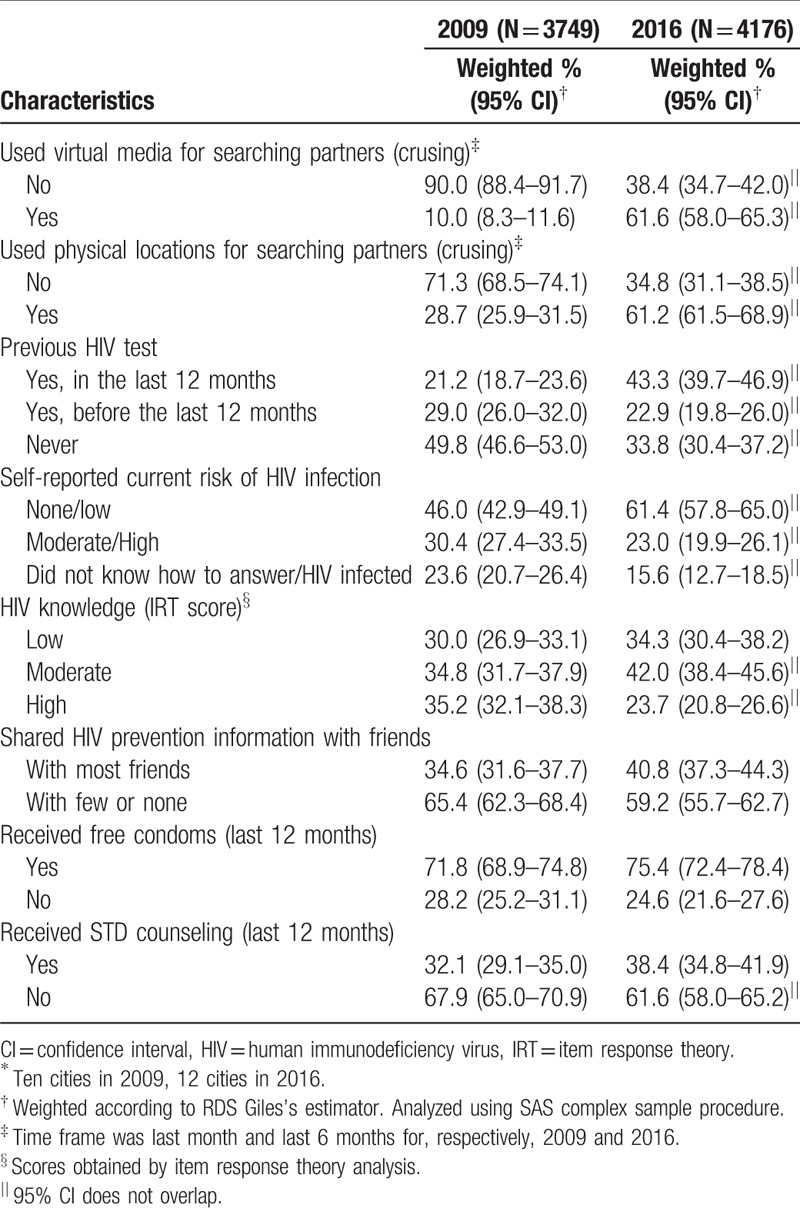
Selected sociobehavior characteristics among 2 cross-sectional MSM RDS studies in Brazil^∗^, 2009 and 2016.

A worrisome picture emerged with regard to sexual behavior characteristics. Despite a reduction in the proportion of early sex debut (<15 years old) from 2009 to 2016 (45.9% vs 40.5%, CI overlapping) and an increase in condom use in the first sex (30.4% vs 45.4%), there was an also increase in sex with 6 or more partners in the past 6 months (25.3–30.5%) while unprotected anal receptive and insertive sex remained at very high rates in 2016 (36.4% and 42.2%, respectively). However, the proportion of stable partnership increased from 51.8% to 68.9% and exchanging sex for money (commercial partnership) dropped from 29.7% to 14.4%, from 2009 to 2016, respectively. We should also note the steep rise in cruising for both, virtual media (10.0% vs 61.6%) and physical locations (28.7% vs 61.2%).

There was a decrease in the proportion of MSM who had never tested for HIV, from 49.8% in 2009, to 33.8%, in 2016. We also note the increase in the proportion of those who assessed their risk of acquiring HIV infection as low or none from 46.0%, in 2009, to 61.4%, in 2016, while there was a decrease in the proportion of those with high HIV knowledge from 35.2%, in 2009, to 23.7%, in 2016. Finally, sharing knowledge with friends was more common in 2016, and, although more than two-thirds of this population received free condoms in both years, mainly from health services, the proportion of those receiving STI counseling remained very low (32.1% and 38.4% for 2009 and 2016, respectively, CI overlapping).

**Figure 1:**
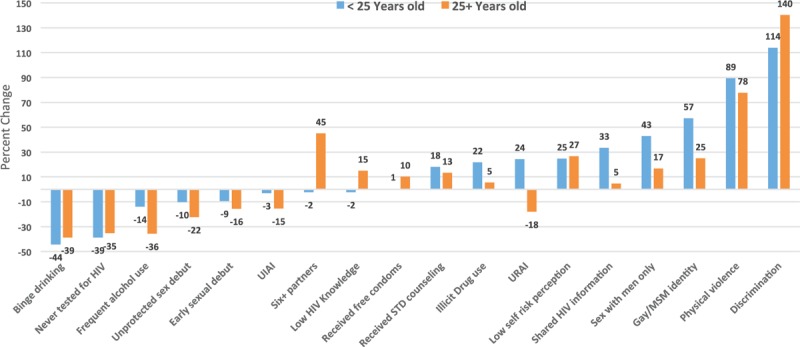
Percent change of selected characteristics from 2009 to 2016, among MSM in Brazil, according to age group.

Further comparisons of twenty selected indicators stratified by age group (<25 and 25+ years old) provide additional data for better understanding behavior trends among MSM in Brazil (Table 2, Fig. 1). Most variables showed percent changes in the same direction for both groups when comparing 2009 and 2016, with some variations in magnitude. On the improvement side, having never been tested for HIV, alcohol use, binge drinking, and early sexual debut (<15 years old) showed negative percent changes from 2009 to 2016, with more pronounced decreases of alcohol use and binge drinking among those 25 years old or older. There were also similar increases in receiving STI counseling for both age groups. Other sexual behaviors demonstrated a trend to riskier sex. For instance, although the proportion of first unprotected sex was lower among those <25 years old, the decrease from 2009 to 2016 in this age group was less pronounced than among those 25+ years old (percent change = −10% and −22%, respectively). Having 6 or more sexual partners remained stable among younger MSM at about a quarter of the population, while among older MSM there was a 45% increase in multiple partnership. There was a decrease in the proportion of unprotected insertive anal intercourse (UIAI) among both age groups, but this was more pronounced among those older MSM (percent change = −3% and −15%, for respectively, <25 and 25+ years old groups). On the contrary, unprotected receptive anal intercourse increased from 2009 to 2016 by 24% and decreased by 18% among younger and older MSM, respectively. Other indicators showed increases for both age groups with varying magnitudes from 2009 to 2016, including low self-reported risk, gay/homosexual identity, physical and sexual violence, discrimination (Table 2).

**Table 2:**
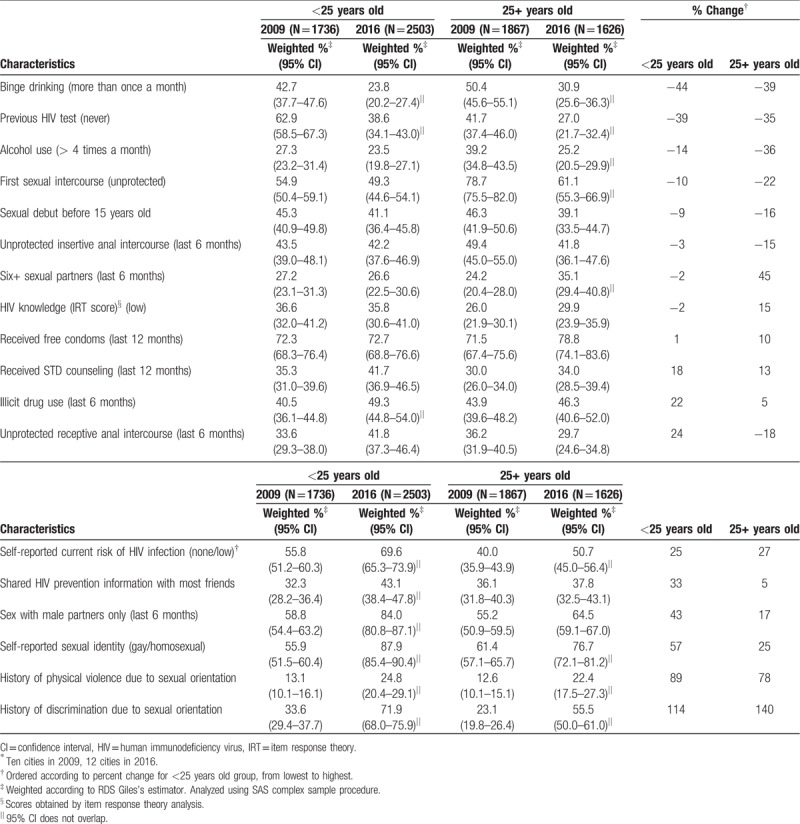
Selected sociobehavior characteristics among 2 cross-sectional MSM RDS Studies in Brazil^∗^, 2009 and 2016, by age group.

Finally, our data indicate a great degree of heterogeneity when comparing selected sexual behaviors across different host cities, as depicted in Table 3. In this case we focused on the 2016 study results only. For instance, sexual debut before 15 years old varied from 35.3% in Belo Horizonte to 52.7% in Manaus; unprotected sexual debut varied from 45.4% in Porto Alegre to a high of 63.6% in Recife; multiple partnership varied from 16.9% in Belém to 36.1% in São Paulo; and unprotected receptive anal intercourse in the previous 6 months varied from 24.8% in Recife to 53.3% in Belém. Other indicators can be seen in Table 3.

**Table 3:**
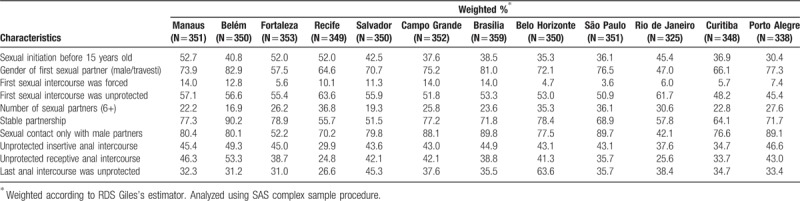
Selected sexual behavioral characteristics of the sample according to host sites, 2016 (N = 4176).

## 4 Discussion

Results from these 2 BBSS surveys among MSM in Brazil are both disturbing and contradictory. On one hand, we show a decrease in alcohol use, an increase in condom use during their first sex, a probably increased awareness of their sexual identity as gay/homosexuals, increased proportion of MSM in stable relationships, and a decreased proportion of exchange sex for money, especially among younger MSM. On the other hand, our data indicate that risk taking practices are increasing, by having more sex with multiple partners, not using condom in both insertive and receptive anal sex, especially among young MSM, with lower levels of counseling uptake and knowledge of HIV information, and increase in illicit drug use. Similar trends among young MSM have been observed in other countries^[28]^ and remains to be further explored. Possible explanations include excessive optimism with treatment options, wide availability of new social media technology, a generation gap associating prevention concerns with older MSM, poor assessment of transmission risks, and poor knowledge about HIV and the consequences of lifetime infection and medication, among others.

As shown, the high proportion of participants who reported receiving free condoms in both years is in contrast to a decrease in the proportion of those with high HIV knowledge, an increase in self-reported low or no risk of acquiring HIV, and low proportions of counseling uptake in both years. This may indicate that condom distribution is probably not being accompanied by counseling, which may have a direct effect on knowledge and risk perception. In addition, although HIV testing has improved, testing without proper counseling may actually reinforce medicalized interventions with potential reduced effectiveness of prevention efforts in Brazil.^[11,12,15,28–31]^

Of great concern are the steep rises in discrimination and violence suffered due to sexual orientation from 2009 to 2016 among MSM in Brazil, mirroring disturbing reports from around the world (UNAIDS, 2016). To what extent greater visibility, reverses in human rights initiatives, or both, contribute to this situation remains to be further analyzed.^[15,28]^

A fuller understanding of these changes needs to be further investigated in up-coming analyses where explanatory variables and interaction of these factors can be properly assessed. From both, a strategic and practical point of view, public health policy makers must not overlook these results. The probable consequences of declining support for prevention and enhanced risk taking include a potential increase in the prevalence of HIV and other STI among MSM in Brazil.^[32]^

## 5 Limitations

First, these are 2 cross-sectional samples of MSM originated from 10 cities in 2009 and 12 cities in 2016, with only 8 cities overlapping in both study rounds. As shown, heterogeneity among the cities may affect the aggregated estimates. Also, when comparing age, schooling and socioeconomic status, the 2016 study population is younger, with better educated and higher socioeconomic status. However, these factors should have contributed to improved prevention behavior, contrary to our findings. Nevertheless, these socioeconomic indicators should be considered for statistical adjustments in future analyses. Thus, although interpretations must proceed with caution, we believe that the objective of the exercise was achieved, and that we produced important information of a rising HIV epidemic among young MSM, and identified some of the behaviors associated with this increase. Finally, methodological issues related to IRT for assessing HIV knowledge and RDS sampling could potentially affect our estimates, as discussed elsewhere.^[21,24,25]^ However, because we used the same methods in both years, and an RDS experienced team participated in the 2 survey rounds, we believe we have reduced some potential sources of errors for the comparison.

In conclusion, our analysis reveals a worrisome picture with regard to behavior among MSM in Brazil, comparing 2009 to 2016, with an apparent increase in HIV risk-related behaviors. These results, alongside increasing prevalence, can greatly contribute to developing new strategies and programs to meet UNAIDS and the DIAHV/MoH goals.

## Author contributions

**Conceptualization:** M.A.R.K.D.C. Guimaraes.

**Formal analysis:** M.A.R.K.D.C. Guimaraes.

**Writing – original draft:** M.A.R.K.D.C. Guimaraes, C. Kendall, L. Magno, G.M. Rocha, D.R. Knauth, I. Dourado, M.A. Veras, L. Kerr.

**Writing – review & editing:** M.A.R.K.D.C. Guimaraes, C. Kendall, L. Magno, G.M. Rocha, D.R. Knauth, I. Dourado, M.A. Veras, A.M. Brito, L. Kerr.

## References

[1] Joint United Nations Programme on HIV/AIDS. UNAIDS Data 2017. Geneva, Switzerland, 2017.

[2] Brazil. Ministry of Health. Health Surveillance Secretariat. Department of STI, AIDS and Viral Hepatitis. [UNGASS–HIV/Aids, Brazilian Answer 2008–2009. Country Progression Report]. Brasília; 2010. Portuguese.

[3] Kerr LR Mota RS Kendall C HIV among MSM in a large middle-income country. AIDS 2013;27:427–35. 23291540 10.1097/QAD.0b013e32835ad504

[4] Guimarães MDC Carneiro M Abreu DMX HIV/AIDS Mortality in Brazil, 2000–2015: are there reasons for concern? Rev Bras Epidemiol 2017;20 (suppl 1):182–90. 10.1590/1980-5497201700050015 28658382

[5] Beyrer C Baral SD Van Griensven F Global epidemiology of HIV infection in men who have sex with men. Lancet 2012;380:367–77. 22819660 10.1016/S0140-6736(12)60821-6 PMC3805037

[6] Baral S Sifakis F Cleghorn F Elevated risk for HIV infection among men who have sex with men in low- and middle-income countries 2000–2006: a systematic review. PLoS Med 2007;4:1901–11. 10.1371/journal.pmed.0040339 PMC2100144 18052602

[7] Sullivan PS Carballo-Dieguez A Coates T Successes and challenges of HIV prevention in men who have sex with men. Lancet 2012;380:388–99. 22819659 10.1016/S0140-6736(12)60955-6 PMC3670988

[8] Baggaley RF White RG Boily MC HIV transmission risk through anal intercourse: systematic review, meta-analysis and implications for HIV prevention. Int J Epidemiol 2010;39:1048–63. 20406794 10.1093/ije/dyq057 PMC2929353

[9] Rocha GM Kerr LR de Brito AM Unprotected receptive anal intercourse among men who have sex with men in Brazil. AIDS Behav 2013;17:1288–95. 23325375 10.1007/s10461-012-0398-4

[10] Centers for Disease Control and Prevention (CDC). Vital signs: HIV infection, testing, and risk behaviors among youths—United States. MMWR Morb Mortal Wkly Rep 2012;61:971–6. 23190571

[11] Balaji AB Bowles KE Le BC High HIV incidence and prevalence and associated factors among young MSM, 2008. AIDS 2013;27:269–78. 23079807 10.1097/QAD.0b013e32835ad489 PMC5098328

[12] Traynor SM Brincks AM Feaster DJ Correlates of unknown HIV status among MSM participating in the 2014 American Men's Internet Survey (AMIS). AIDS Behav 2017;https://doi.org/10.1007/s10461-017-1894-3 https://doi.org/10.1007/s10461-017-1894-3. [Epub ahead of print]. 10.1007/s10461-017-1894-3 PMC6870175 28852893

[13] Wong VJ Murray KR Phelps BR Adolescents, young people, and the 90-90-90 goals: a call to improve HIV testing and linkage to treatment. AIDS 2017;31 (suppl 3):S191–4. 28665876 10.1097/QAD.0000000000001539 PMC5497776

[14] Brazil. Ministry of Health. Secretary of Health Surveillance. Department of STI, AIDS and Viral Hepatitis. Boletim Epidemiológico–Aids e DST. 2017; 3–77 [Portuguese]

[15] Malta M, Beyrer C. The HIV epidemic and human rights violations in Brazil. J Int AIDS Soc 2013; 16: 18817 10.7448/IAS.16.1.18817 PMC3827457 24225350

[16] Joint United Nations Programme on HIV/AIDS. Monitoring the Declaration of Commitment on HIV/AIDS: guidelines on construction of core indicators: 2010 reporting. Geneva, Switzerland, 2009

[17] Alfven T Erkkola T Ghys PD Global AIDS reporting—2001 to 2015: lessons for monitoring the sustainable development goals. AIDS Behav 2017;21 (suppl 1):5–14. 28124296 10.1007/s10461-016-1662-9 PMC5515967

[18] Mills S Saidel T Bennett A HIV risk behavioral surveillance: a methodology for monitoring behavioral trends. AIDS 1998;12 (suppl 2):37–46. 9792360

[19] Rehle T Lazzari S Dallabetta G Second generation HIV surveillance: better data for decision-making. Bull World Health Organ 2004;82:121–7. 15042234 PMC2585900

[20] Barbosa Junior A Pascom AR Szwarcwald CL Transfer of sampling methods for studies on most-at-risk populations (MARPs) in Brazil. Cad Saude Publica 2011;27 (suppl 1):S36–44. 21503522 10.1590/s0102-311x2011001300005

[21] Kendall C Kerr LR Gondim RC An empirical comparison of respondent-driven sampling, time location sampling, and snowball sampling for behavioral surveillance in men who have sex with men, Fortaleza, Brazil. AIDS Behav 2008;12 (suppl 4):S97–104. 18389357 10.1007/s10461-008-9390-4

[22] Kendall C Kerr LR Mota RS The 12 city HIV surveillance survey among MSM in Brazil 2016 using respondent-driven sampling: a description of methods and RDS diagnostics. Rev Bras Epidemiol 2017;submitted. 10.1590/1980-549720190004 30892467

[23] ABEP. Associação Brasileira de Empresas e Pesquisa. Critério Brasil 2015 e atualização da distribuição de classes para 2016. Available at: http://www.abep.org/criterio-brasil. Accessed on Oct 6, 2017.

[24] Gomes RRMF Batista JR Ceccato MGB HIV/AIDS knowledge among men who have sex with men: applying the item response theory. Rev Saúde Pública 2014;48:206–15. 24897041 10.1590/S0034-8910.2014048004911 PMC4206150

[25] Gomes RRFM Ceccato MGB Kerr LRFS Factors associated with low knowledge on HIV/AIDS among men who have sex with men in Brazil. Cad Saúde Pública 2017;33:e00125515 http://dx.doi.org/10.1590/0102-311x00125515 29091176 10.1590/0102-311X00125515

[26] Gile KJ Handcock MS Respondent-driven sampling: an assessment of current methodology. Sociol Methodol 2010;40:285–327. 22969167 10.1111/j.1467-9531.2010.01223.x PMC3437336

[27] Handcock MS, Fellows IE, Gile KJ. 2016 RDS Analyst: Software for the Analysis of Respondent-Driven Sampling Data. Version 0.57, Available at: http://hpmrg.org. Accessed September 29, 2017.

[28] Beyrer C Sullivan P Sanchez J The increase in global HIV epidemics in MSM. AIDS 2013;27:2665–78. 23842129 10.1097/01.aids.0000432449.30239.fe

[29] Witzel TC Hickson F Weatherburn P HIV testing history and preferences for future tests among gay men, bisexual men and other MSM in England: results from a cross-sectional study. BMJ Open 2016;6:e011372. 10.1136/bmjopen-2016-011372 PMC5030541 27630068

[30] Chan PA Towey C Poceta J Online hookup sites for meeting sexual partners among men who have sex with men in Rhode Island, 2013: a call for public health action. Public Health Rep 2016;131:264–71. 26957661 10.1177/003335491613100210 PMC4765975

[31] Mcdaid LM Aghaizu A Frankis J Frequency of HIV testing among gay and bisexual men in the UK: implications for HIV prevention. HIV Med 2016;17:683–93. 26991460 10.1111/hiv.12373 PMC5026165

[32] Kerr L Kendall C Guimarães MDC HIV prevalence among men who have sex with men in a middle-income country: results of the second national survey using respondent-driven sampling in Brazil. Medicine/HIV 2017;submitted. 10.1097/MD.0000000000010573 PMC5991534 29794604

